# Atypical Flutter with Atrial Isochronal Late-activation Map Correlating with the Critical Isthmus

**DOI:** 10.19102/icrm.2021.120108S

**Published:** 2021-01-15

**Authors:** Sunil Kapur

**Affiliations:** ^1^Brigham and Women’s Hospital, Boston, MA, USA

**Keywords:** Atrial isochronal late-activation mapping, atypical atrial flutter, critical isthmus

Ablation of atypical atrial flutter is an increasingly challenging and prevalent problem. However, while induction of the arrhythmia is fundamental to the ablation strategy, this may not be universally possible. As an alternative, ultra–high-density mapping during sinus rhythm allows for the creation of isochronal late-activation maps (ILAMs) in patients with ventricular tachycardia and facilitates the identification of a critical isthmus even without induction of the ventricular arrhythmia. Creating an ILAM of the left atrium has not been systematically evaluated and it is not known whether additional ablation in these areas improves freedom from all atrial arrhythmias.

We report a case of left atrial mapping performed using the Advisor™ HD Grid Mapping Catheter, Sensor Enabled™ in a patient referred for atypical atrial flutter ablation after prior pulmonary vein isolation. The arrhythmia could not be induced at the start of the case; subsequently, a left atrial map with high right atrial pacing was created and we identified a deceleration zone on the anterior left atrium **(Figure 1 and Video 1)**. Subsequent induction of the arrhythmia was possible and activation mapping suggested the previously identified region was the critical isthmus. Ablation in this region terminated the tachycardia. This case supports the validity of atrial ILAM as a strategy for the empiric ablation of atypical atrial flutter.

## Figures and Tables

**Figure 1:**
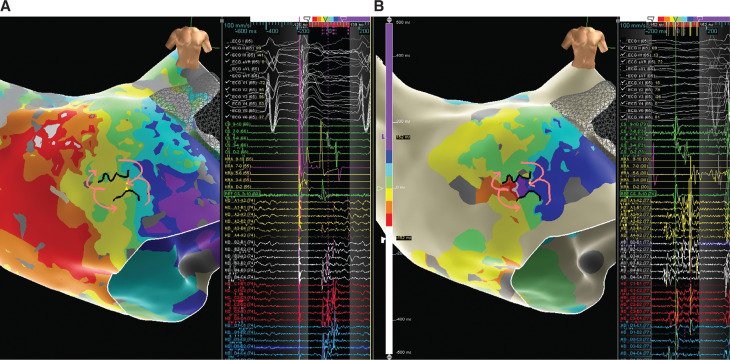
**A:** Atrial ILAM with right atrial pacing. **B:** ILAM of induced tachycardia.

**Video 1.** Atypical flutter with atrial ILAM correlating with the critical isthmus.

